# Epigenetic regulation of interleukin-8 expression by class I HDAC and CBP in ovarian cancer cells

**DOI:** 10.18632/oncotarget.19990

**Published:** 2017-08-07

**Authors:** Himavanth R. Gatla, Yue Zou, Mohammad M. Uddin, Ivana Vancurova

**Affiliations:** ^1^ Department of Biological Sciences, St. John’s University, New York City, NY 11439, USA

**Keywords:** interleukin-8, HDAC, CBP, ovarian cancer

## Abstract

Although inhibitors of epigenetic regulators have been effective in the treatment of cutaneous T cell lymphoma (CTCL) and other hematopoietic malignancies, they have been less effective in solid tumors, including ovarian cancer (OC). We have previously shown that inhibition of histone deacetylase (HDAC) activity induces expression of the pro-inflammatory and pro-angiogenic chemokine interleukin-8 (CXCL8, IL-8) in OC cells, resulting in their increased survival and proliferation. Here, we show that in addition to ovarian cancer SKOV3, OVCAR3, and CAOV3 cells, HDAC inhibition induces the CXCL8 expression in HeLa cells, but not in CTCL Hut-78 cells. In OC cells, the CXCL8 expression is induced by pharmacological inhibition of class I HDACs. Interestingly, while an individual suppression of HDAC1, HDAC2, or HDAC3 by corresponding siRNAs inhibits the CXCL8 expression, their simultaneous suppression induces the CXCL8 expression. The induced CXCL8 expression in OC cells is dependent on histone acetyltransferase (HAT) activity of CREB-binding protein (CBP), but not p300, and is associated with HAT-dependent p65 recruitment to CXCL8 promoter. Together, our results show that the CXCL8 expression in OC cells is induced by combined inhibition of HDAC1, -2, and -3, and silenced by suppression of HAT activity of CBP. In addition, our data indicate that the induced CXCL8 expression may be responsible for the limited effectiveness of HDAC inhibitors in OC and perhaps other solid cancers characterized by CXCL8 overexpression, and suggest that targeting class I HDACs and CBP may provide novel combination strategies by limiting the induced CXCL8 expression.

## INTRODUCTION

Ovarian cancer (OC), the most common cause of cancer deaths among gynecological malignancies, is characterized by an increased expression of the pro-inflammatory and pro-angiogenic chemokine interleukin-8 (CXCL8, IL8) [1]. The increased release of CXCL8 in OC cells increases their survival, proliferation, and migration, thus contributing to OC metastasis and angiogenesis [2-5]. At the transcriptional level, the CXCL8 expression in OC cells is regulated by IκB kinase (IKK)-dependent p65 NFκB promoter recruitment [5, 6]; both IKK and p65 NFκB activities are constitutively increased in OC, and convey a poor outcome [7-9]. The promoter accessibility is regulated by reversible histone acetylation, which is controlled by two opposing enzymes: histone acetyltransferases (HATs) and histone deacetylases (HDACs) [10].

Based on their function and sequence homology, HDACs are grouped into four classes, class I-IV. Class I HDACs, which include HDAC1, 2, 3, and 8, are increased in OC cells, and their overexpression is associated with poor prognosis and development of chemoresistance [11-13]. The FDA-approved pan-HDAC inhibitor vorinostat (suberoylanilide hydroxamic acid, SAHA) and class I HDAC inhibitor romidepsin (depsipeptide, FK228) have been remarkably effective in the treatment of cutaneous T cell lymphoma (CTCL) and other hematological malignancies [14-19]. In contrast, HDAC inhibitors (HDIs) as single agents have been far less effective in the treatment of OC and other solid tumors, but the reasons are not fully understood. Recent studies from our laboratory have shown that vorinostat induces CXCL8 expression in OC cells, and that the induced CXCL8 expression increases OC cell survival, suggesting that the induced CXCL8 release might represent one of the underlying mechanisms responsible for the limited effectiveness of HDAC inhibitors in OC [5, 20]. However, the specific HDAC involvement in the regulation of CXCL8 expression in OC cells remains unknown.

CREB-binding protein (CBP) and p300 are HATs that share a high degree of homology and are responsible for acetylation of histones as well as non-histone proteins [21-25]. CBP and p300 are not only important epigenetic factors but also key regulators of cell differentiation and carcinogenesis; however, in contrast to HDAC inhibitors, the potential use of CBP/p300 inhibitors as anticancer agents has received considerably less attention, particularly because of the lack of specific inhibitors [26, 27]. The first selective p300/CBP inhibitor developed is the pyrazolone benzoic acid C646, which has exhibited anti-proliferative and pro-apoptotic properties in several types of cancer cells, including leukemia, melanoma, pancreatic and prostate cancer [28-31]. However, the role of CBP/p300 HATs in the CXCL8 regulation in OC cells is unknown.

Since the HDAC inhibition by vorinostat has been very effective in the treatment of CTCL [14-19], in this study, we have investigated whether vorinostat induces the CXCL8 expression in CTCL Hut-78 cells, as well as in the widely used cervical cancer HeLa cells. In addition, we have analyzed the specific involvement of HDACs and p300/CBP in the epigenetic regulation of CXCL8 expression in OC cells. Our results show that in addition to OC cells, HDAC inhibition by vorinostat induces CXCL8 expression in HeLa cells, but not in CTCL Hut-78 cells. In addition, our data demonstrate that the CXCL8 expression in OC cells is specifically induced by a combined inhibition of HDAC 1, 2, and 3, and silenced by an inhibition of CBP HAT activity, suggesting that targeting HDAC 1, 2, and 3, and CBP may provide novel combination strategies in OC by limiting the increased CXCL8 expression.

## RESULTS

### HDAC inhibition by vorinostat induces CXCL8 expression in OC and HeLa cells, but not in CTCL Hut-78 cells

**Figure 1:**
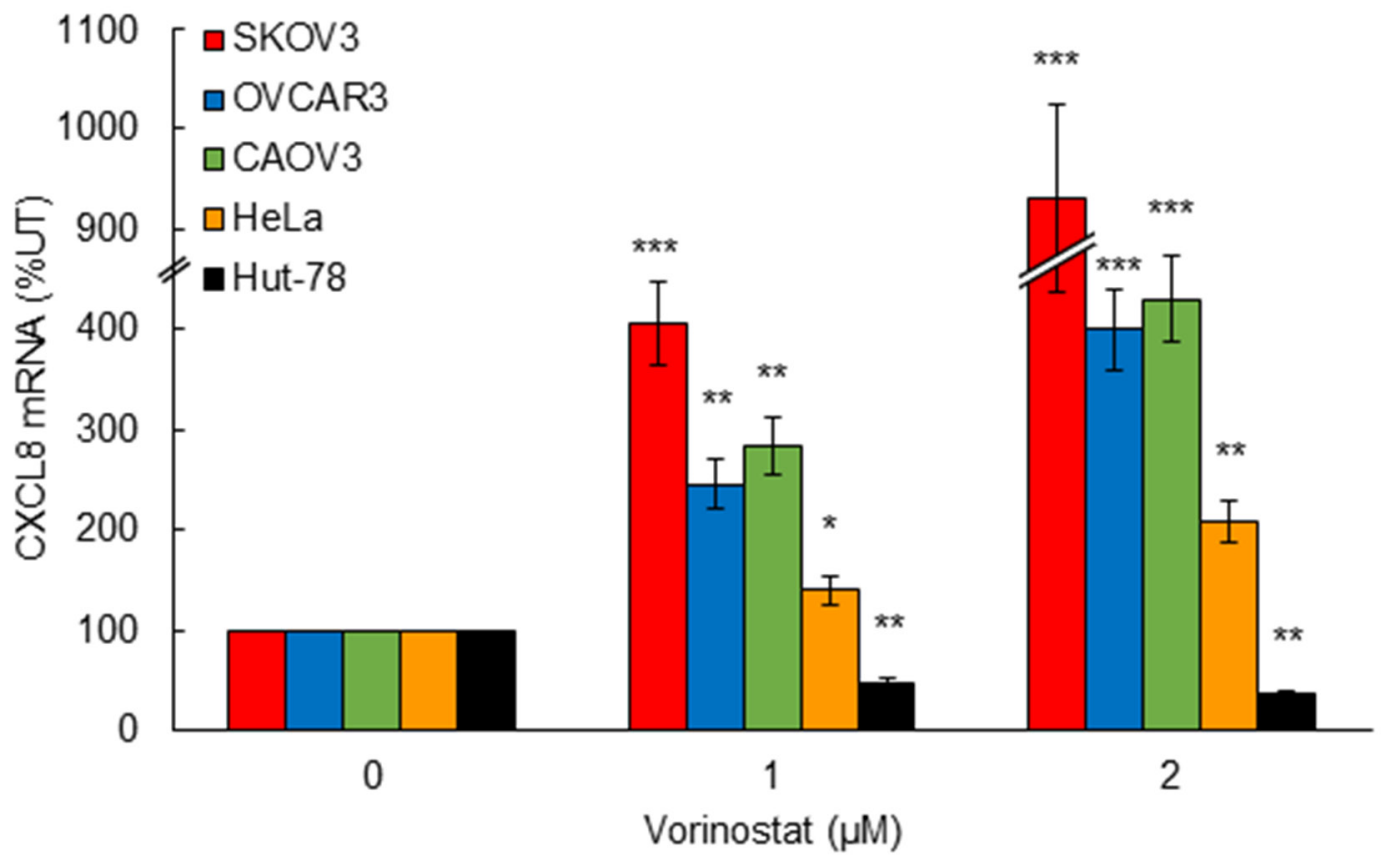
HDAC inhibition by vorinostat induces CXCL8 expression in OC and HeLa cells, but not in CTCL Hut-78 cells Real time RT-PCR analysis of CXCL8 mRNA levels in SKOV3, OVCAR3, CAOV3, HeLa, and Hut-78 cells incubated with increasing vorinostat concentrations for 48 hours. The values represent the mean +/-SE of three experiments; asterisks denote a statistically significant change compared to untreated control cells.

Despite the limited effectiveness of HDAC inhibitors as a single agent therapy in ovarian cancer and other solid tumors, the HDAC inhibitors vorinostat and romidepsin have been remarkably effective in the treatment of CTCL [14-19]. Since we have recently shown that HDAC inhibition specifically induces CXCL8 expression in OC cells, resulting in their increased survival and proliferation [5], we wanted to determine whether HDAC inhibition by vorinostat induces the CXCL8 expression also in CTCL cells, where vorinostat has been effectively used for CTCL treatment. Interestingly, while vorinostat significantly increased the CXCL8 expression in ovarian cancer SKOV3, OVCAR3, and CAOV3 cells, as well as in cervical cancer HeLa cells, the CXCL8 expression in CTCL Hut-78 cells was actually inhibited by 1 and 2 μM vorinostat (Figure 1), which approximately corresponds to the clinically used concentrations [32]. These data suggest that the HDI-induced CXCL8 expression may represent one of the mechanisms responsible for the limited effectiveness of HDAC inhibitors in OC treatment.

### Simultaneous inhibition of HDAC1, 2 and 3 induces CXCL8 expression in OC cells

**Figure 2:**
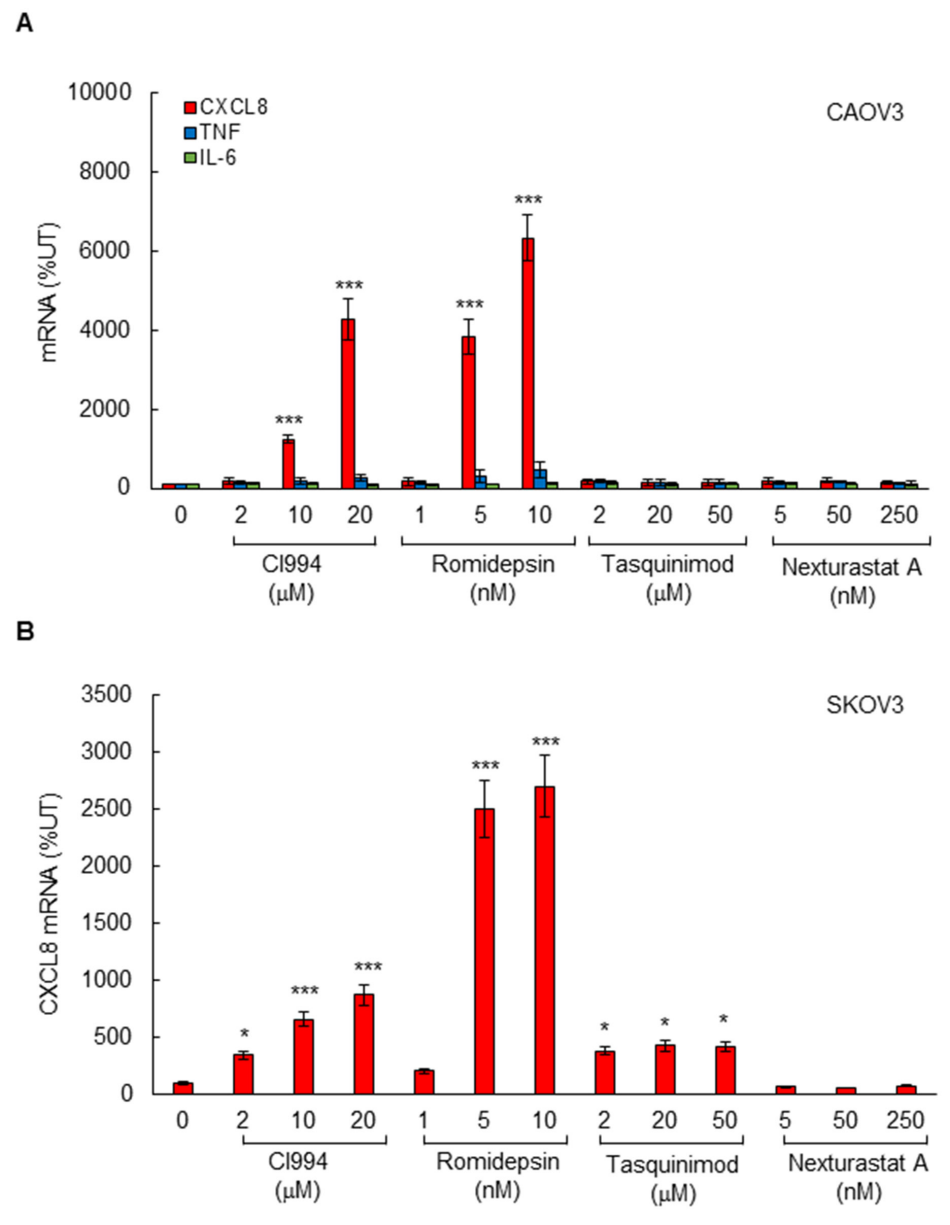
Class I HDAC inhibition induces CXCL8 expression in OC cells **(A)** Real time RT-PCR analysis of mRNA levels of CXCL8, TNFα, and IL-6 in CAOV3 cells incubated with CI994, romidepsin, tasquinimod, or nexturastat A for 48 hours. **(B)** Real time RT-PCR of CXCL8 mRNA levels in SKOV3 cells incubated with CI994, romidepsin, tasquinimod, or nexturastat A for 48 hours. The values represent the mean +/-SE of four experiments; asterisks denote a statistically significant (* p<0.05; ** p<0.01; *** p<0.001) change compared to control untreated (UT) cells.

To determine which HDAC is specifically involved in the HDI-induced CXCL8 expression in OC cells, we first analyzed CXCL8 expression in CAOV3 cells incubated 48 hours with increasing concentrations of different HDAC inhibitors. CI994 and romidepsin are specific class I HDAC inhibitors; CI994 inhibits HDAC1, 2, and 3, while romidepsin preferentially inhibits HDAC1 and HDAC2 [14, 15, 33]. Tasquinimod and nexturastat are class II HDAC inhibitors; tasquinimod inhibits HDAC4 [34], and nexturastat inhibits HDAC6 [35]. As shown in Figure 2A, the CXCL8 expression was specifically induced by class I HDAC inhibitors, CI994 and romidepsin, but not by class II inhibitors, tasquinimod and nexturastat. As we have previously observed [5], the robust induction of NFκB-dependent cytokine expression was specific for CXCL8, since HDAC inhibition did not induce mRNA levels of other NFκB-dependent cytokines, TNFα and IL-6 (Figure 2A). The CXCL8 expression was significantly induced by class I HDAC inhibitors also in SKOV3 cells; however, in SKOV3 cells, the CXCL8 expression was induced also by the HDAC4 inhibitor tasquinimod (Figure 2B).

**Figure 3:**
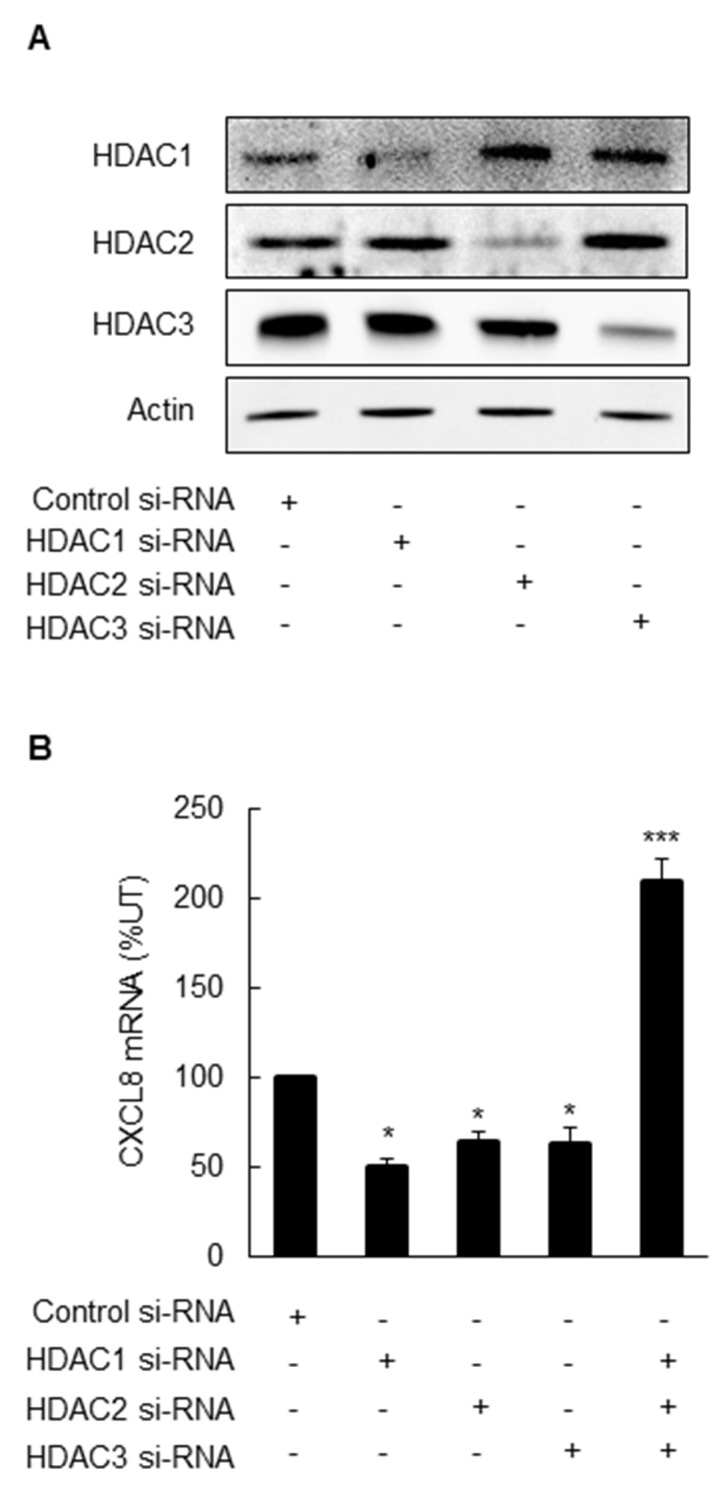
Simultaneous suppression of HDAC1, 2 and 3 induces CXCL8 expression in OC cells **(A)** Western blotting analysis of HDAC1, 2, 3, and control actin levels in whole cell extracts prepared from SKOV3 cells transfected with control, HDAC1, 2, or 3 specific siRNAs. Each lane corresponds approximately to 5x10^4^ cells. **(B)** Real-time RT-PCR analysis of CXCL8 mRNA levels in SKOV3 cells transfected with control and HDAC1, 2, and 3 specific siRNAs alone or in combination, as described above. The values represent the mean +/-SE of four experiments; asterisks denote a statistically significant change compared to control UT cells.

To confirm the pharmacological inhibition results and analyze the roles of the individual class I HDACs in the CXCL8 regulation in OC cells, we analyzed CXCL8 expression in SKOV3 cells transfected with HDAC1, -2, and -3 siRNAs. Transfection with HDACs specific siRNAs reduced their corresponding protein levels, demonstrating specificity of the transfections (Figure 3A). Surprisingly however, in contrast to the pharmacological inhibition that increased the CXCL8 expression, silencing the individual HDAC1, -2, or -3 decreased the CXCL8 mRNA levels in OC cells (Figure 3B). Simultaneous suppression of HDAC1, -2, and -3 induced the CXCL8 expression (Figure 3B); however, this increase was considerably lower compared to the pharmacological inhibition by class I HDAC inhibitors (Figure 2).

### HDI-induced CXCL8 expression is dependent on HAT activity of p300/CBP in OC cells

**Figure 4:**
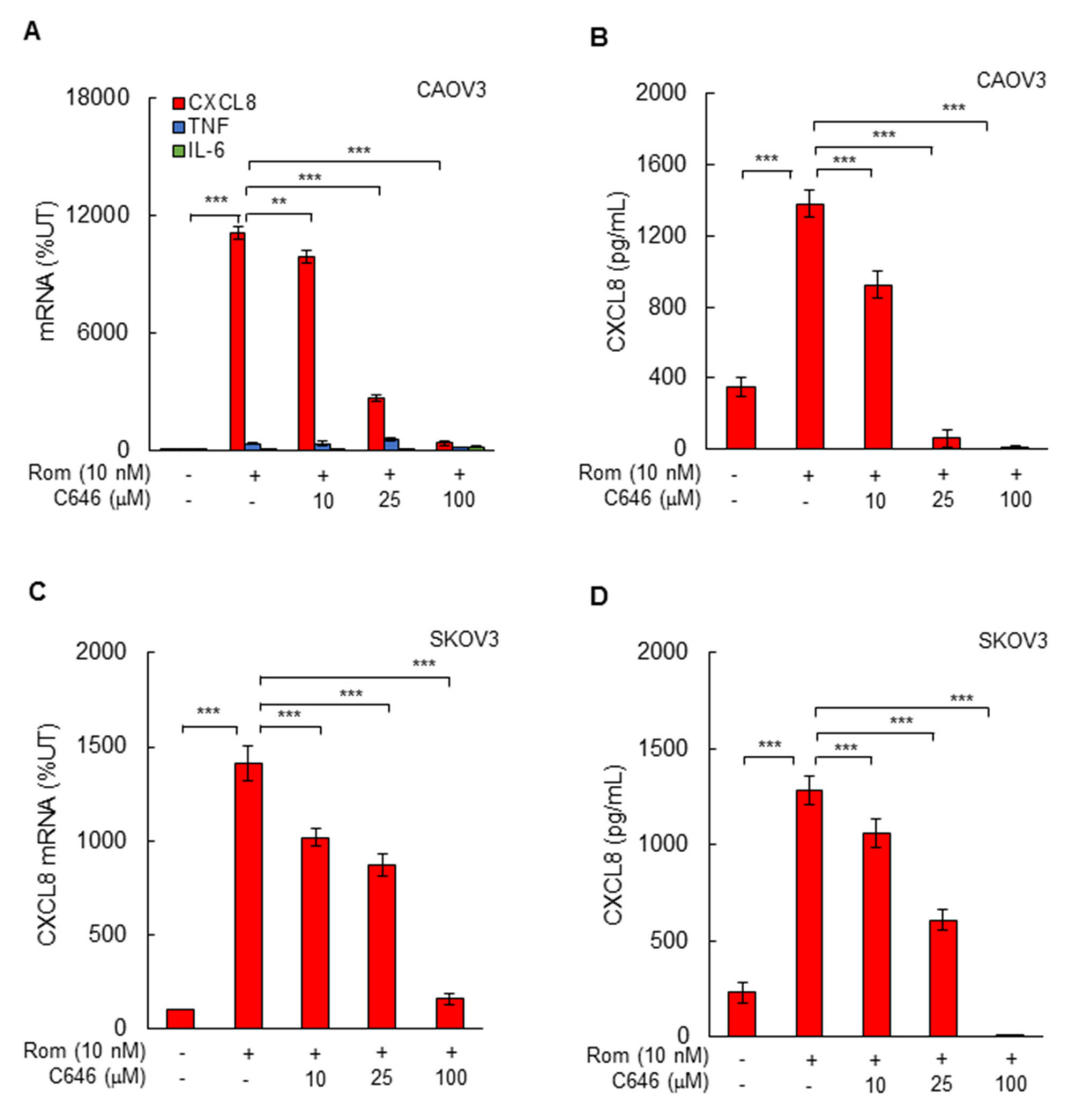
Romidepsin-induced CXCL8 expression in OC cells is dependent on HAT activity **(A)** Real-time RT-PCR of mRNA levels of CXCL8, TNFα, and IL-6, and **(B)** ELISA of CXCL8 release in CAOV3 cells pre-treated with increasing C646 concentrations for 12 h, followed by incubation with 10 nM romidepsin for 48 h. **(C)** RT-PCR of CXCL8 mRNA levels, and **(D)** ELISA of CXCL8 release in SKOV3 cells pre-incubated with C646 and treated with romidepsin as described above. The values represent the mean +/-SE of four experiments; asterisks denote a statistically significant change compared to UT cells, or cells treated with romidepsin alone.

To determine whether the HDI-induced CXCL8 expression in OC cells is dependent on HAT activity of p300/CBP, we analyzed the CXCL8 mRNA and protein levels in CAOV3 and SKOV3 cells incubated with 10 nM romidepsin, in the presence of the selective p300/CBP inhibitor C646 [28-31]. Inhibition of p300/CBP HAT activity significantly reduced the romidepsin-induced CXCL8 mRNA levels and cytokine release both in CAOV3 (Figure 4A, 4B) and SKOV3 (Figure 4C, 4D) cells, demonstrating that the HDI-induced CXCL8 expression is dependent on the p300/CBP HAT activity.

### Suppression of CBP inhibits CXCL8 expression in OC cells

**Figure 5:**
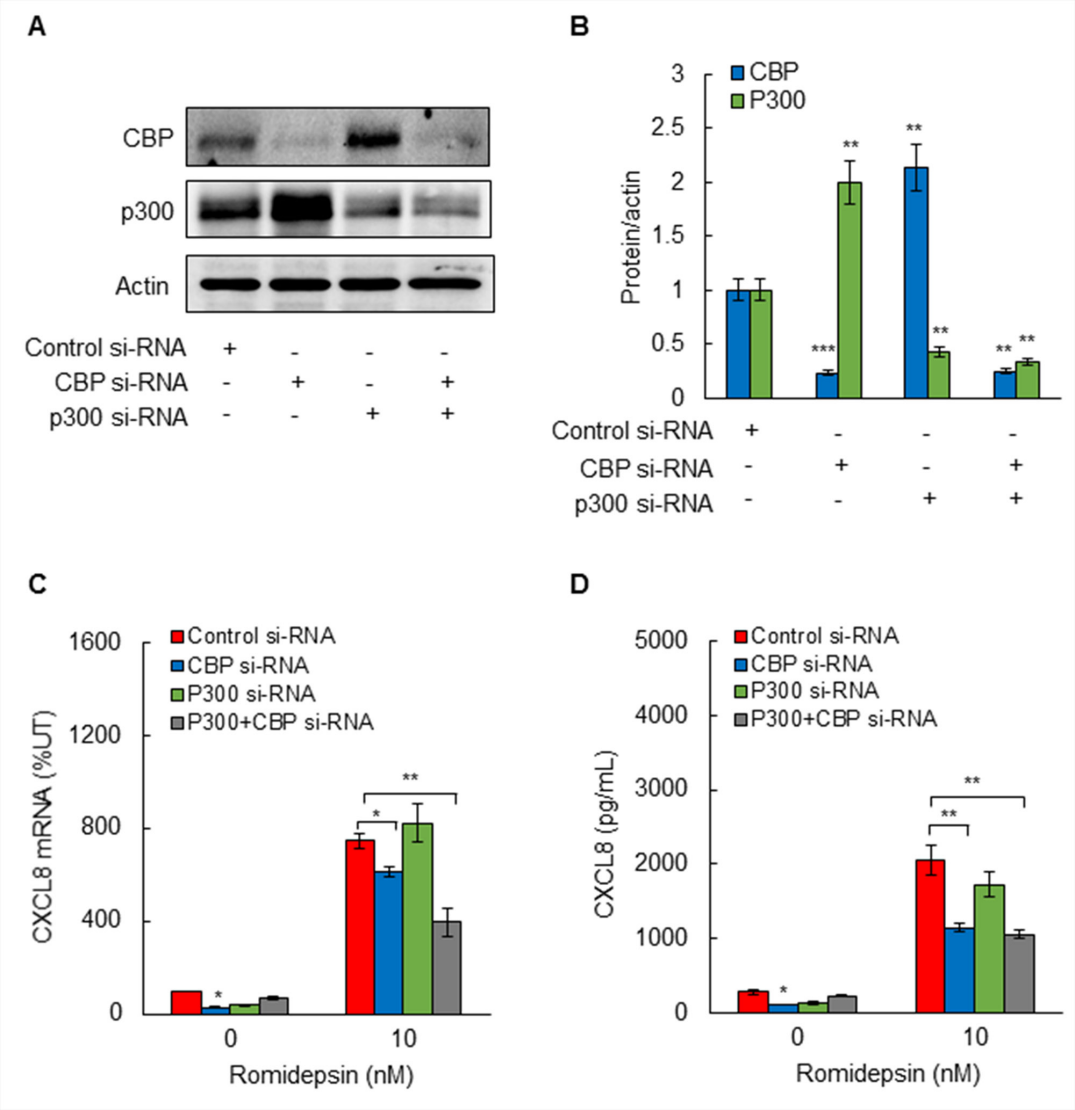
Suppression of CBP inhibits romidepsin-induced CXCL8 expression in OC cells **(A)** Western blotting analysis of CBP, p300, and control actin levels in whole cells extracts prepared from SKOV3 cells transfected with control siRNA, or siRNA specific for p300 or CBP, alone and in combination. Each lane corresponds approximately to 5x10^4^ cells. **(B)** Densitometric evaluation of CBP and p300 protein levels shown in panel A, normalized to actin. The values for control siRNAs were arbitrarily set to 1, and the other values are presented relative to these values. **(C)** RT-PCR of CXCL8 mRNA levels; and **(D)** ELISA of CXCL8 release in SKOV3 cells transfected with control, CBP, and/or p300 siRNA, and treated 48 h with 10 nM romidepsin or control DMSO. The values represent the mean +/-SE of four experiments; asterisks denote a statistically significant change compared to cells treated with control siRNA.

Since C646 inhibits the HAT activity of both p300 and CBP, we wanted to determine whether p300 and/or CBP activity is involved in the HDI-induced CXCL8 expression in OC cells. SKOV3 cells were transfected with CBP and p300 specific siRNAs, both individually and in combination. Compared to control siRNA, transfection with CBP or p300 siRNA significantly suppressed the corresponding protein levels of both HATs (Figure 5A, 5B). Interestingly however, suppression of p300 increased the protein level of CBP, and suppression of CBP increased the protein levels of p300 (Figure 5A, 5B), indicating that suppression of one HAT upregulates expression of the other HAT. Importantly, suppression of CBP, but not p300, inhibited the CXCL8 mRNA expression (Figure 5C) and cytokine release (Figure 5D), indicating that only CBP mediates the HDI-induced CXCL8 expression in OC cells.

### CBP HAT activity mediates p65 NFκB recruitment to CXCL8 promoter in OC cells

**Figure 6:**
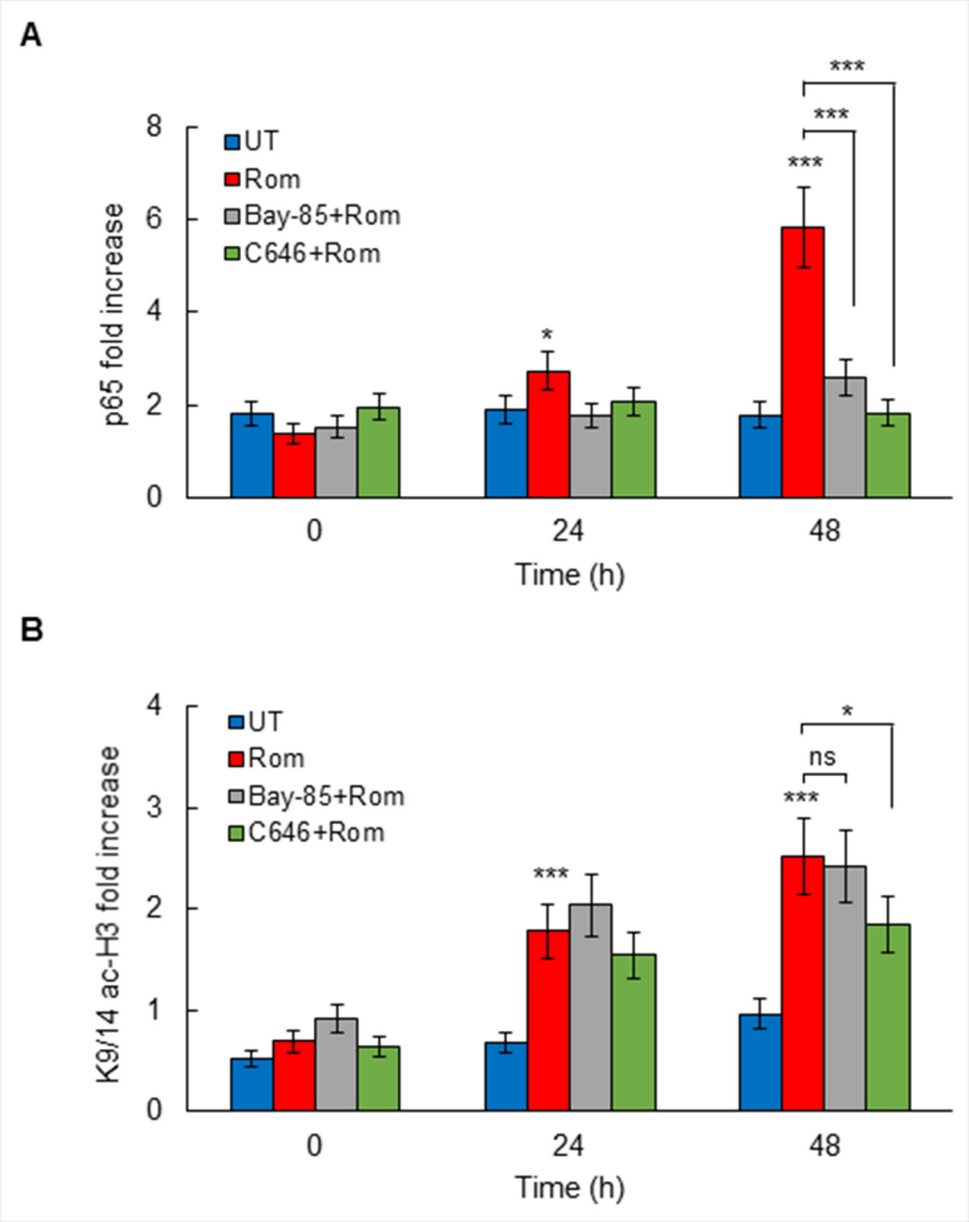
Romidepsin induces HAT-dependent p65 and K9/14 ac-H3 occupancy at CXCL8 promoter ChIP analysis of p65 occupancy **(A)** and K9/14 ac-H3 occupancy **(B)** at CXCL8 promoter quantified by real-time PCR, in SKOV3 cells pre-treated 12 h with 5 μM Bay-118075, 25 μM C646, or control DMSO, and incubated with 10 nM romidepsin. The results are presented as a fold difference in occupancy over the human IGX1A (SA Biosciences) sequence control, and represent the mean +/-SE of three experiments; asterisks denote a statistically significant change compared to control UT cells.

To determine whether the CBP HAT activity regulates p65 NFκB recruitment to CXCL8 promoter in OC cells, we analyzed p65 occupancy at the CXCL8 promoter in SKOV3 cells incubated 0, 24, and 48 hours with 10 nM romidepsin, in the absence and presence of IKK inhibitor, Bay 117085 (5 μM), or C646 (25 μM). Corresponding with the induced CXCL8 expression, HDAC inhibition with romidepsin significantly increased p65 recruitment to CXCL8 promoter (Figure 6A). The romidepsin-induced p65 occupancy at the CXCL8 promoter at 48 h was inhibited by Bay 117085, indicating that the HDI-induced p65 promoter occupancy is dependent on IKK activity, as we have previously observed in vorinostat-treated OC cells [5]. Importantly, the induced p65 promoter occupancy at 48 h was also inhibited by C646 (Figure 6A), indicating that the CBP HAT activity is required for p65 recruitment.

In addition, the HDAC inhibition by romidepsin induced occupancy of K9/14 acetylated histone H3 (K9/14 ac-H3) at CXCL8 promoter (Figure 6B). The K9/14 ac-H3 promoter occupancy preceded the p65 occupancy, indicating that HDAC inhibition induces first histone H3 acetylation, thus enabling p65 recruitment. The K9/14 ac-H3 promoter occupancy at 48 h was inhibited by C646, but not Bay 117085 (Figure 6B), suggesting that the K9/14 ac-H3 promoter occupancy is dependent on HAT, but not IKK activity.

## DISCUSSION

**Figure 7:**
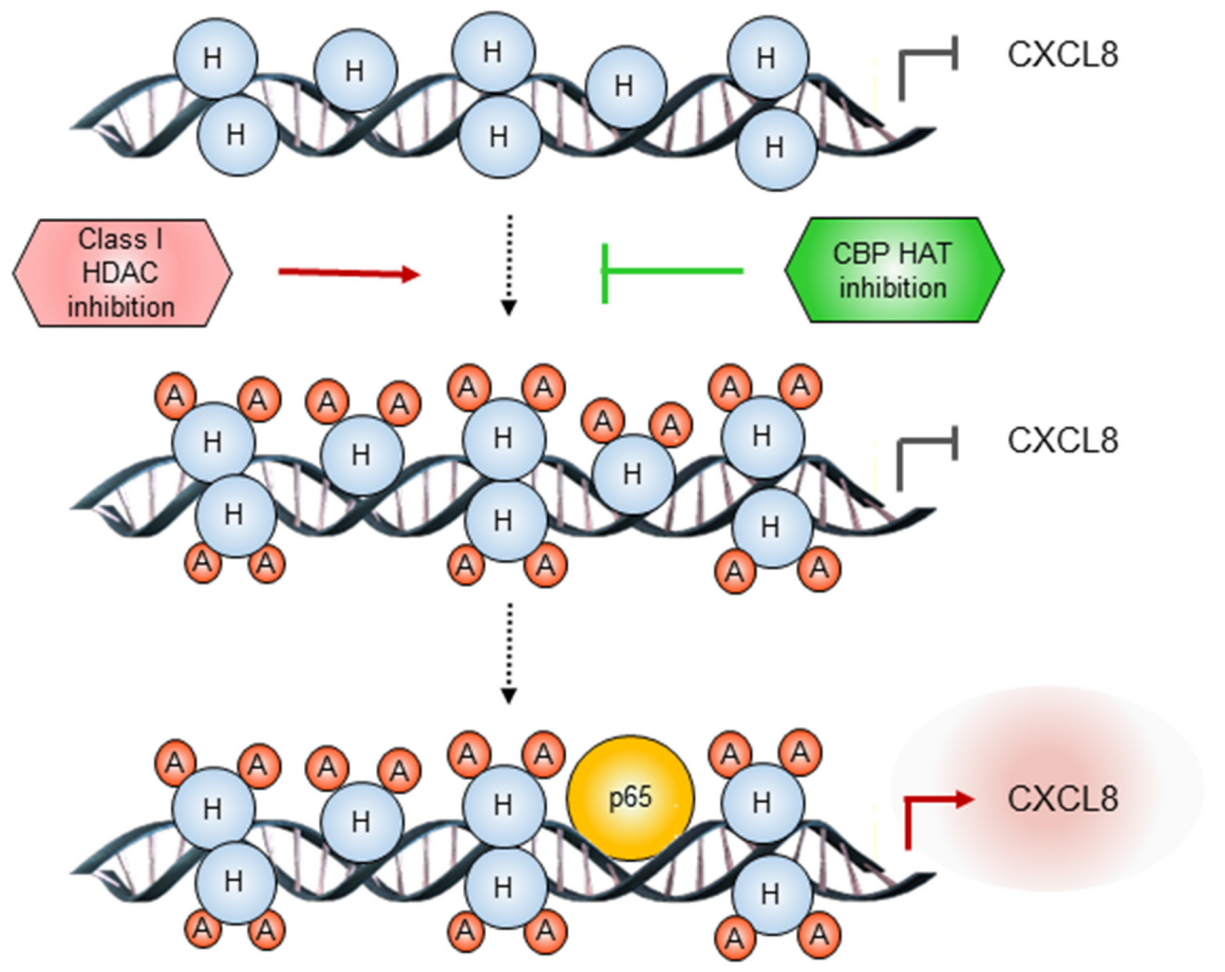
Proposed model of CXCL8 regulation by class I HDACs and CBP in OC cells In OC cells, inhibition of class I HDAC activity increases acetylation of histone H3 at Lys 9/14, thus facilitating p65 recruitment to CXCL8 promoter. Inhibition of CBP HAT activity decreases the histone acetylation, resulting in decreased p65 recruitment, and decreased CXCL8 transcription.

Inhibitors of epigenetic regulators have demonstrated great promise in hematopoietic malignancies, but have been less effective in solid tumors, including ovarian cancer. Since CXCL8 mediates proliferation of OC cells, and is associated with poor outcome in OC patients [1-5], in this study, we have investigated the specific involvement of HDACs and HATs in the epigenetic regulation of CXCL8 expression in OC cells. The key findings of this study are that the CXCL8 expression in OC cells is induced by simultaneous inhibition of HDAC1, 2, and 3, and suppressed by inhibition of CBP HAT activity. Our results support a model in which class I HDAC inhibition increases CBP-dependent K9/14 ac-H3 promoter occupancy, which is followed by increased p65 recruitment to CXCL8 promoter, resulting in increased CXCL8 transcription in OC cells (Figure 7). These data provide the first evidence indicating that the CXCL8 expression in OC cells is regulated by CBP and suggesting that targeting the HAT activity of CBP may increase effectiveness of HDAC inhibitors in OC treatment by decreasing the CXCL8 transcription.

Expression of HDAC1, -2, and -3 is increased in OC tissues compared to normal ovarian tissues, and correlates with poor prognosis [11, 36-38]. However, compared to CTCL and other hematological cancers, targeting class I HDAC activity in OC, as well as in other solid tumors, has been ineffective. HDAC inhibitors have been developed as anti-cancer agents for their ability to induce hyperacetylation of histones and non-histone proteins, resulting in increased apoptosis and differentiation of cancer cells [14, 15]. Remarkably, our results demonstrate that HDAC inhibition by vorinostat induces CXCL8 expression in OC and HeLa cells, but not in CTCL Hut-78 cells. Since CXCL8 overexpression promotes tumor cell proliferation and angiogenesis, these data suggest that compared to CTCL cells, the induced CXCL8 expression in OC cells may represent one of the mechanisms responsible for the limited effectiveness of HDAC inhibitors in OC and other solid tumors characterized by the increased CXCL8 expression.

Interestingly, in contrast to the pharmacological inhibition, individual suppression of HDAC1, -2, and -3 by siRNA did not increase the CXCL8 expression in OC cells, but inhibited it. These results indicate that HDAC-1, -2, and -3 may form protein complexes with other transcriptional regulators that control the CXCL8 transcription in OC cells, and suppression of HDAC protein levels in these complexes disrupts their function and reduces the CXCL8 transcription. In this context, previous studies have shown that HDAC1, -2, and -3 form complexes with p65 NFκB, IκBα, NuRD, N-CoR/SMRT, and other transcriptional activators and repressors important for cancer cell survival and growth [39-44]. Thus, the impact of pharmacologic inhibition of HDACs will likely differ from the effect of HDAC protein suppression. This is further supported by our data demonstrating that even though the simultaneous suppression of HDAC1, -2, and -3 induced the CXCL8 expression, indicating that HDAC1, -2, and -3 function together in suppressing the CXCL8 transcription in OC cells; it was to a much lower extent compared to the pharmacological inhibition.

The p300 and CBP histone acetyltransferases promote transcription by functioning as transcriptional adaptors between enhancer-bound transcription factors and the basal transcription apparatus, as well as by acetylating more than 70 different proteins, including themselves [45-47]. Even though p300 and CBP share a high degree of homology and redundant overlapping activities, they also have distinct functions that regulate cell cycle and differentiation [26, 27, 48, 49]. Indeed, our study shows that suppression of CBP upregulates the protein levels of p300 in OC cells, and conversely, suppression of p300 upregulates the protein expression of CBP, indicating that suppression of one HAT results in compensatory upregulation of the other HAT. However, only CBP seems to be involved in the HDI-induced CXCL8 transcription in OC cells, since suppression of p300 did not inhibit the romidepsin-induced CXCL8 expression. However, our data indicate that the HAT activity of CBP is essential for the HDI-induced CXCL8 expression in OC cells, since pharmacological inhibition of HAT activity significantly suppressed the CXCL8 expression. The HAT activity is required for both the HDI-induced K9/14 ac-H3 promoter occupancy, and for the subsequent recruitment of p65 to CXCL8 promoter. Since CBP is upregulated in cisplatin-resistant ovarian cancer cells [50], these data suggest that CBP may serve as a potential therapeutic target in OC.

Together, our results demonstrate that the CXCL8 expression in OC cells is induced by simultaneous inhibition of HDAC1, -2, and -3, and suppressed by inhibition of CBP HAT activity, suggesting that targeting class I HDACs and CBP may provide novel combination strategies for OC treatment by limiting the increased CXCL8 expression.

## EXPERIMENTAL PROCEDURES

### Antibodies and reagents

Antibodies against human p65 NFκB (sc-372), K9/14 acetylated histone H3 (sc-8655), HDAC1 (sc-81598), HDAC2 (sc-6296), HDAC3 (sc-11417), CBP (sc-7300), and p300 (sc-585) were purchased from Santa Cruz Biotechnology (Santa Cruz, CA). Antibody against actin was purchased from Sigma (St. Louis, MO). Horseradish peroxidase (HRP)-conjugated secondary antibodies were from Santa Cruz Biotechnology (Santa Cruz, CA). Romidepsin was from ChemieTek (Indianapolis, IN). CI994, tasquinimod, and nexturastat A were from Selleckchem (Houston, TX). IKK inhibitor Bay 117085 was purchased from Cayman Chemicals (Ann Arbor, MI). All other reagents were molecular biology grade and were from Sigma (St. Louis, MO).

### Cell culture

Human SKOV3, CAOV3, OVCAR3, HeLa, and Hut-78 cells were obtained from the American Type Culture Collection (ATCC; Rockville, MD). Cells were cultured (5x10^5^ cells/ml) in 6-well plates in RPMI 1640 medium (Invitrogen, Grand Island, NY) supplemented with 10 % heat inactivated fetal bovine serum (FBS; Invitrogen, Grand Island, NY) and antibiotics at 37℃ with 5% CO_2_ as described [5, 6]. The pharmacological inhibitors were dissolved in DMSO, and an equivalent DMSO volume was used as a solvent control.

### Transfection with siRNA

Human HDAC1 (sc-29343), HDAC2 (sc-29345), HDAC3 (sc-35538), p300 (sc-29431), CBP (sc-29244), and non-silencing (sc-37007) small interfering RNAs (siRNAs) were obtained from Santa Cruz Biotechnology. Prior to transfection, cells were seeded into a 6-well plate and incubated in a humidified 5% CO_2_ atmosphere at 37°C in antibiotic-free RPMI medium supplement with 10% FBS for 24 hours to about 80% confluence. For each transfection, 80 pmol of either non-silencing siRNA control or specific siRNA were used. Cells were transfected 7 hours in siRNA transfection medium with siRNA transfection reagent according to manufacturer’s instructions (Santa Cruz Biotechnology). After transfection, fresh medium with antibiotics was added, and cells were grown for 24 hours before treatment.

### Real time RT-PCR

Total RNA was isolated by using RNeasy mini-kit (Qiagen, Valencia, CA). The iScript one-step RT-PCR kit with SYBR Green (Bio-Rad, Hercules, CA) was used as a supermix and 20 ng/μl of RNA was used as template on a Bio-Rad MyIQ Single Color Real-Time PCR Detection System (Bio-Rad). The primers used for quantification of human IL-8/CXCL8, TNFα, IL-6, and actin mRNA were purchased from SA Biosciences (Frederick, MD). The mRNA values are expressed as a percentage of untreated (UT) samples, which were arbitrarily set as 100% [5, 6].

### Western analysis

Whole cell extracts were prepared as described previously [51, 52]. Denatured proteins were separated on 10% -14% SDS gels, and analyzed by immunoblotting as described [51, 52].

### ELISA

Human CXCL8 release was measured by commercially available ELISA kit (R&D, Minneapolis, MN) as described [5, 6].

### Chromatin immunoprecipitation (ChIP)

ChIP analysis was performed as described [5, 6]. Briefly, proteins and DNA were cross-linked by formaldehyde, and cells were washed and sonicated. The lysates were centrifuged (15,000 *g,* 10 min, 4 °C), and the supernatant extracts were diluted with ChIP dilution buffer and pre-cleared with Protein A/G Agarose (Santa Cruz, CA) for 2 hours at 4 °C. Immunoprecipitations were performed overnight at 4 °C. Following immunoprecipitation, the samples were incubated with Protein A/G Agarose (1 h, 4 °C), and the immune complexes were collected by centrifugation (150 *g*, 5 min, 4 °C), washed, and extracted with 1% SDS–0.1 M NaHCO_3_. After reversing the cross-linking, proteins were digested with proteinase K, and the samples were extracted with phenol/chloroform, followed by precipitation with ethanol. The pellets were re-suspended in nuclease-free water and subjected to real time PCR. Immunoprecipitated DNA was analyzed by real-time PCR (25 μl reaction mixture) using the iQ SYBR Green Supermix and the Bio-Rad MyIQ Single Color Real-Time PCR Detection System (Bio-Rad). Each immunoprecipitation was performed at least three times using different chromatin samples, and the occupancy was calculated by using the human IGX1A negative control primers (SA Biosciences, Frederick, MD), which detect specific genomic ORF-free DNA sequence that does not contain binding site for any known transcription factors. The results were calculated as fold difference in occupancy of the particular protein at the particular locus in comparison with the IGX1A locus. The CXCL8 primers used for real time PCR were: forward, 5’-GGGCCATCAGTTGCA-AATC-3’ and reverse, 5’-GCTTGTGTGCTCT-GCTGTCTC-3’.

### Statistical analysis

The results represent at least three independent experiments. Numerical results are presented as means ± SE. Data were analyzed by using an InStat software package (GraphPAD, San Diego, CA). Statistical significance was evaluated by using Mann-Whitney U test, and p<0.05 was considered significant. Levels of significance are indicated as *p<0.05; **p<0.01; and ***p<0.001.

## Abbreviations

ChIP: chromatin immunoprecipitation
OC: ovarian cancer
HAT: histone acetyltransferase
HDAC: histone deacetylase
HDI: histone deacetylase inhibitor
IKK: IκB kinase
CTCL: cutaneous T cell lymphoma
